# Summarizing health-related quality of life (HRQOL): development and testing of a one-factor model

**DOI:** 10.1186/s12963-016-0091-3

**Published:** 2016-07-11

**Authors:** Shaoman Yin, Rashid Njai, Lawrence Barker, Paul Z. Siegel, Youlian Liao

**Affiliations:** SciMetrika, LLC, 100 Capitola Drive, Durham, NC 27701 USA; Centers for Disease Control and Prevention, 4770 Buford Highway NE, Atlanta, GA 30341 USA

**Keywords:** Health-related quality of life, Summary score, Factor analysis

## Abstract

**Background:**

Health-related quality of life (HRQOL) is a multi-dimensional concept commonly used to examine the impact of health status on quality of life. HRQOL is often measured by four core questions that asked about general health status and number of unhealthy days in the Behavioral Risk Factor Surveillance System (BRFSS). Use of these measures individually, however, may not provide a cohesive picture of overall HRQOL. To address this concern, this study developed and tested a method for combining these four measures into a summary score.

**Methods:**

Exploratory and confirmatory factor analyses were performed using BRFSS 2013 data to determine potential numerical relationships among the four HRQOL items. We also examined the stability of our proposed one-factor model over time by using BRFSS 2001–2010 and BRFSS 2011–2013 data sets.

**Results:**

Both exploratory factor analysis and goodness of fit tests supported the notion that one summary factor could capture overall HRQOL. Confirmatory factor analysis indicated acceptable goodness of fit of this model. The predicted factor score showed good validity with all of the four HRQOL items. In addition, use of the one-factor model showed stability, with no changes being detected from 2001 to 2013.

**Conclusion:**

Instead of using four individual items to measure HRQOL, it is feasible to study overall HRQOL via factor analysis with one underlying construct. The resulting summary score of HRQOL may be used for health evaluation, subgroup comparison, trend monitoring, and risk factor identification.

**Electronic supplementary material:**

The online version of this article (doi:10.1186/s12963-016-0091-3) contains supplementary material, which is available to authorized users.

## Background

Health-related quality of life (HRQOL) is a useful indicator of overall health because it captures information on the physical and mental health status of individuals, and on the impact of health status on quality of life [1, 2]. HRQOL is usually assessed via multiple indicators of self-perceived health status and physical and emotional functioning. Together, these measures provide a comprehensive assessment of the burden of preventable diseases, injuries, and disabilities [3].

To assess and measure HRQOL at the state and national levels, the Centers for Disease Control and Prevention (CDC) developed a set of four “core” questions (CDC HRQOL-4): (1) Would you say that in general your health is excellent, very good, good, fair, or poor? (2) Now thinking about your physical health, which includes physical illness and injury, for how many days during the past 30 days was your physical health not good? (3) Now thinking about your mental health, which includes stress, depression, and problems with emotions, for how many days during the past 30 days was your mental health not good? (4) During the past 30 days, for about how many days did poor physical or mental health keep you from doing your usual activities, such as self-care, work, or recreation? [3–5].

These four items, which have demonstrated good retest reliability, validity, and responsiveness [6–8], have been included in the Behavioral Risk Factor Surveillance System (BRFSS) in all 50 states since 1993. In addition, the four items have also been included in other national surveys (e.g., National Health and Nutrition Examination Survey (NHANES), Medicare Health Outcome Survey) and in various chronic disease assessments [7, 9, 10]. CDC HRQOL-4 account for similar variance as the Patient-Reported Outcome Measurement Information System (PROMIS) items (e.g., SF-36) [11–13]. However, the CDC items appear more appropriate for assessing burden of disease for chronic conditions and are brief and easily interpretable [11].

In 1995, CDC added five additional questions related to quality of life to BRFSS, as part of an optional module. The new questions asked about days experiencing pain, feeling sad or depressed, feeling worried or anxious, not getting enough rest, or feeling healthy. However, the optional module was only used in a limited number of states and years.

To assess HRQOL comprehensively, public health professionals have sought a means to summarize these HRQOL measures. To combine the information on physically and mentally unhealthy days, some researchers have summed the two measures in CDC HRQOL-4 to create an Unhealthy Days Index, with the sum of the two items being truncated at 30 days [3, 14, 15]. This approach assumes an independent relationship between the two kinds of days.

Another approach is to view HRQOL as a latent (hidden) construct that can be quantified through factor analysis. Factor analysis is a method for detecting relationships among variables, which often reduces the number of variables. Previous studies found strong associations among the CDC HRQOL-4 questions, suggesting that these items may be suitable for factor analysis [4]. Toet and colleagues found good internal consistency of the four measures (the Cronbach’s alpha for the three unhealthy day measures was 0.77; a Cronbach’s alpha of 0.70 or more is usually considered acceptable [16]) [13]. Horner-Johnson and colleagues, on the other hand, found a relatively poor consistency between the mentally unhealthy day item and the three other items based on “the Cronbach’s alpha increase if item removed” test [17]. They compared two alpha values: one based on all items; the other based on remaining items after a test item was removed. This analysis relies on the premise that if the test item value increases, this may indicate poor consistency of the removed item. However, due to the lack of a clear cutoff value for the increase, it is a somewhat subjective choice to remove a single item measure, especially for situations in which the increase in the alpha values is minimal. Horner-Johnson and colleagues found only a very slight increase (e.g., 0.001 when using BRFSS 2002 data), which may not be enough to undermine the internal consistency of the mentally unhealthy day item with other HRQOL items [17]. Raykov and colleagues warned that the Cronbach’s alpha if item is removed test can be misleading for selecting construct components [18, 19].

Two studies have conducted HRQOL factor analysis using the CDC HRQOL-4 plus the five optional HRQOL module questions [7, 17]. Using data from BRFSS (2001 and 2002), both studies demonstrated that the nine HRQOL questions have good internal consistency and could be reduced to two latent factors that correspond to the physical and mental health aspects of HRQOL. However, data from the optional BRFSS module were only available for a few states and years, which limits the application of these models in tracking HRQOL over the years or assessing HRQOL at the national level.

This study proposes a method for creating a summary score of overall HRQOL based solely on CDC HRQOL-4. Public health professionals could treat such a consolidated score as a “new” variable that could be used to describe both community and population health, assess health disparities, monitor trends, and identify risk factors of overall HRQOL at the local and/or national levels. Using the 2013 BRFSS data set, the study assesses whether there is an underlying latent construct of HRQOL for the general population, and investigates the possibility of reducing CDC HRQOL-4 to one summary score. It also provides an example of how this type of summary score could be used in trend analysis using BRFSS 2001–2010 and 2011–2013 data sets.

## Methods

### Data sources

The BRFSS is a state-based random-digit-dialed telephone health survey system. The survey annually collects data from non-institutionalized civilian adults (≥18 years of age) about their health-related risk behaviors, chronic health conditions, and use of preventive services [20]. Starting in 2011, BRFSS changed its weighting methodology and added cellular telephone users to its samples. Due to these changes, caution should be used when comparing BRFSS data from before and after 2011 [21]. In our analyses, we included two groups of data sources: BRFSS 2013 data (as an experimental study for factor analysis) and BRFSS 2001–2013 data sets (to assess model stability and perform trend analysis, one for 2001–2010 data sets and another for 2011–2013 data sets). Data on the four HRQOL questions were available from all states for every year, except 2002, when data were available from 22 states only.

### Statistical analysis

To study the underlying structure of the CDC HRQOL-4, we conducted Cronbach’s alpha test, exploratory factor analysis (EFA), and confirmatory factor analysis (CFA) using BRFSS 2013 data. We then assessed the stability of the resulting model over years, and demonstrated its applications for trend analysis using BRFSS 2001–2010 and 2011–2013 data sets.

To analyze the internal consistency or reliability of the CDC HRQOL-4, we performed Cronbach’s alpha test (a larger alpha value indicates greater internal correlation). We used the traditional cutoff value of 0.70 or higher as being acceptable [16]. To reveal construct dimensions, EFA was used, with factors with an eigenvalue (a number showing how much variance there is for that underlying factor) larger than or equal to 1.0 being considered acceptable [22]. The principal axis factoring with rotation of orthogonal varimax rotation was used, which can accommodate non-normal data distribution [23].

Based on the results of Cronbach’s alpha test and EFA, we hypothesized that it would be possible to summarize the CDC HRQOL-4 items by using a single factor. To determine if the model adequately fit the data, we conducted a goodness of fit test using CFA. We used an asymptotically distribution-free method to account for non-normality of the data and ordinal data [24]. Five model fit statistics were used to evaluate model fit: root mean squared error of approximation (RMSEA), comparative fit index (CFI), Tucker-Lewis index (TLI), standardized root mean squared residual (SRMR), and coefficient of determination (CD). We followed commonly accepted criteria regarding goodness of fit: RMSEA (≤0.06), CFI and/or TLI (≥0.95), SRMR (≤0.08), and CD close to 1 [25]. Using one-factor model regression, we generated HRQOL factor score values. To confirm the validity of the HRQOL factor scores, we compared the mean changes in the HRQOL factor scores with each level of the HRQOL measures.

After establishing the one-factor HRQOL model using BRFSS 2013 data, we assessed model stability over the years using two data sets: BRFSS 2001–2010 (10 years) and 2011–2013 (3 years). To do so, we conducted a series of hierarchical tests including factorial configural invariance (similar factor structure across groups), metric invariance (equivalent factor loadings across groups), and scalar invariance (equivalent intercepts across groups) [26]. In sequencing of these tests (increasing constraints on model parameters), we followed the recommended criteria, which suggest that the more restrictive nested model with a decrease of CFI less or equal to 0.01 be accepted [27, 28]. Next, HRQOL factor scores for the 13 years were generated by model predication. Survey sampling design and weighting were considered in the analyses. The year 2000 US standardized population was used for age standardization. All analyses were conducted using STATA 13.0 statistical software (College Station, TX: StataCorp LP).

## Results

### Factor structure

Using BRFSS 2013 data, we first analyzed the correlation matrix and internal consistency of the CDC HRQOL-4 questions (Table 1). The Cronbach’s alpha value of the CDC HRQOL-4 was 0.76, which was within the acceptable range [16]. The alpha change if the item were removed test indicated good consistency within items. Removing the mentally unhealthy day items increased alpha by 1.3 %, which is consistent with Horner-Johnson’s results [6]. EFA (Table 2) showed that a single factor, with an eigenvalue larger than one, explained 99.9 % of the total variance. Therefore, we propose a one-factor HRQOL model for the CDC HRQOL-4.

**Table 1:** Correlation matrix and internal consistency of the CDC HRQOL-4 items, BRFSS 2013

	General health status	Physically unhealthy days	Mentally unhealthy days	Activity limitation days	Cronbach's alpha if item removed	Change (%)
General health status^a^	1				0.733	-3.9
Physically unhealthy days^b^	0.52	1			0.651	-14.6
Mentally unhealthy days^c^	0.29	0.35	1		0.773	+1.3
Activity limitation days^d^	0.43	0.65	0.44	1	0.656	-14
Overall construct					0.763	

^a^Would you say that in general your health is excellent, very good, good, fair, or poor?

^b^Now thinking about your physical health, which includes physical illness and injury, for how many days during the past 30 days was your physical health not good?

^c^Now thinking about your mental health, which includes stress, depression, and problems with emotions, for how many days during the past 30 days was your mental health not good?

^d^During the past 30 days, for about how many days did poor physical or mental health keep you from doing your usual activities, such as self-care, work, or recreation?

**Table 2:** Exploratory factor analysis of the CDC HRQOL-4, BRFSS 2013

Factor	Eigenvalue	Percentage of explained variance	Accumulated Percentage of explained variance
factor 1	1.76	99.9	99.9
factor 2	<0.01	0.1	100.0

### Factor model

**Fig. 1:**
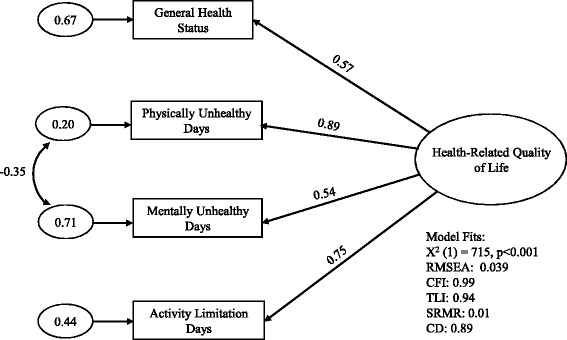
Final one-factor model for the CDC HRQOL-4, BRFSS 2013. Standardized factor loadings from the latent construct (represented by the large oval) to its measures (represented by rectangles) are shown beside the single-headed arrows. The small ovals represent error variances unexplained by the model. The curved double-headed arrow represents correlations between error variances

An initial model with four paths from one factor to the four CDC HRQOL-4 items was first evaluated by CFA. The four items had factor loadings that ranged from 0.46 to 0.87, larger than the minimal acceptable cutoff value of ±0.3 [26]. The goodness of fit statistics indicate that the model is acceptable but could be improved upon (RMSEA = 0.086, CFI = 0.90, TLI = 0.70, SRMR = 0.03, CD = 0.85). To determine whether the model could be improved, a post-hoc model modification was performed. We found that adding an error correlation path between the physically unhealthy day item and the mentally unhealthy day item substantially improved the goodness of fit between model and data. Thus, a final model was proposed (Fig. 1). The minimal factor loading was increased from 0.46 to 0.54. The goodness of fit statistics were also greatly improved (RMSEA = 0.039, CFI = 0.99, TLI = 0.94, SRMR = 0.01, CD = 0.89).

### Factor scores

**Fig. 2:**
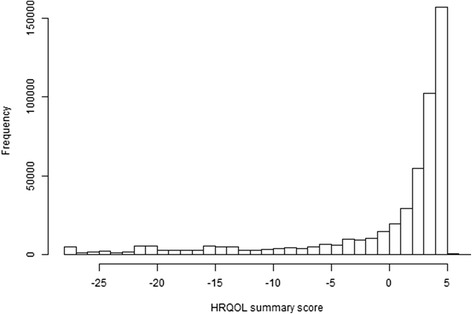
HRQOL summary score, BRFSS 2013. Histogram shows the distribution of the HRQOL summary score using BRFSS 2013 data set. Larger value means better quality of HRQOL. The “skewed left” distribution suggests that the majority of the population is healthy in terms of HRQOL

To quantify the overall HRQOL, weighted factor score values were predicted by the final CFA model. Factor score could be considered as weighted sum scores (multiplying the score of each item into its factor loading and then summing all of them). Figure 2 shows the distribution of predicted factor scores using BRFSS 2013 data, with a larger value indicating better quality of HRQOL. The “skewed left” distribution suggests that the majority of the population is healthy in terms of HRQOL. To check the consistency of HRQOL factor scores with their original measures, we summarized HRQOL factor scores for each level of CDC HRQOL-4 (Table 3). Either in one year or across years, the overall means of HRQOL factor scores decrease as the CDC HRQOL-4 ratings become worse for both male and female adults (we did an analysis stratified by sex, discussed later), indicating the validity of factor scores in representing HRQOL.

**Table 3:** The mean values of HRQOL summary scores by the CDC HRQOL-4 measures

BRFSS Data Sets	2005		2001-2010		2013		2011-2013	
Sex	Male	Female	Male	Female	Male	Female	Male	Female
General Health Status								
Excellent	3.10	4.04	3.14	4.05	3.32	4.17	3.35	4.18
Very good	1.81	2.58	1.84	2.59	2.09	2.84	2.13	2.84
Good	-0.20	0.42	-0.18	0.44	-0.03	0.63	0.01	0.63
Fair	-6.21	-5.60	-6.20	-5.58	-6.55	-5.84	-6.50	-5.84
Poor	-16.91	-16.14	-16.89	-16.12	-17.34	-16.46	-17.27	-16.45
Physically Unhealthy Days								
0 day	2.80	3.50	2.82	3.52	2.99	3.67	3.02	3.67
1-10 days	-0.04	0.71	-0.01	0.73	0.07	0.79	0.11	0.79
11-20 days	-7.98	-7.10	-7.96	-7.08	-7.85	-6.98	-7.79	-6.97
21-29 days	-15.81	-14.72	-15.79	-14.71	-15.64	-14.60	-15.57	-14.57
30 days	-21.17	-19.97	-21.16	-19.96	-20.85	-19.69	-20.77	-19.66
Mentally Unhealthy Days								
0 day	1.11	1.77	1.12	1.80	1.30	2.00	1.34	1.99
1-10 days	-0.24	0.52	-0.22	0.54	-0.31	0.43	-0.26	0.44
11-20 days	-6.09	-5.05	-6.02	-5.06	-6.39	-5.50	-6.32	-5.47
21-29 days	-10.32	-8.97	-10.18	-9.01	-10.91	-9.88	-10.83	-9.82
30 days	-14.79	-13.17	-14.60	-13.23	-15.42	-14.24	-15.31	-14.15
Activity Limitation Days								
0 day	1.58	2.30	1.60	2.32	1.80	2.51	1.84	2.51
1-10 days	-2.00	-1.20	-1.97	-1.19	-1.95	-1.19	-1.90	-1.18
11-20 days	-11.18	-10.19	-11.12	-10.17	-10.93	-10.02	-10.87	-9.99
21-29 days	-17.33	-16.22	-17.24	-16.20	-16.98	-15.97	-16.93	-15.95
30 days	-22.14	-21.01	-22.04	-20.96	-21.57	-20.51	-21.52	-20.49

Summary score with larger value means better quality of HRQOL

### Model stability

To test whether our HRQOL model was stable over time, we examined BRFSS data from 2001 to 2013. Table 4 summarizes the goodness of fit statistics of the model using a series of BRFSS data sets. For all the data sets, whether the combined (2001–2010 or 2011–2013) or individual years were examined, our HRQOL model exhibited acceptable goodness of fit (RMSEA = 0.035-0.05, CFI = 0.984-0.99, TLI = 0.915-0.938, SRMR = 0.01-0.014, and CD = 0.868-0.885). To further examine this, we analyzed results from a sequence of hierarchical tests (Table 5). For both of the combined data sets (2001–2010 and 2011–2013), all models had acceptable goodness of fit statistics (RMSEA = 0.02-0.044, CFI = 0.977-0.987, TLI = 0.925-0.984, SRMR = 0.011-0.014, and CD = 0.879-0.884). The decrease in CFI was no larger than 0.01 for each model pairwise comparison, whether it involved full metric invariance versus full configural invariance, or full scalar invariance versus full metric invariance. These results indicate that the new, single measure of HRQOL has strong measurement invariance, holding full equivalent factor patterns, full equivalent factor loadings, and full equivalence intercepts over the years, from 2001 to 2010, and from 2011 to 2013.

**Table 4:** Summary of fit statistics for the one-factor HRQOL model over time

BRFSS Data Sources	χ^2^	RMSEA	CFI	TLI	SRMR	CD
2001-2010 (10 years)	5441	0.041	0.987	0.924	0.012	0.880
2001	248	0.035	0.989	0.933	0.010	0.868
2002	126	0.036	0.986	0.918	0.011	0.873
2003	319	0.035	0.99	0.937	0.010	0.880
2004	448	0.039	0.988	0.928	0.011	0.881
2005	499	0.038	0.989	0.934	0.011	0.877
2006	519	0.039	0.989	0.933	0.011	0.881
2007	720	0.042	0.988	0.926	0.012	0.883
2008	665	0.041	0.988	0.929	0.011	0.879
2009	793	0.044	0.987	0.920	0.012	0.879
2010	1090	0.050	0.984	0.902	0.014	0.884
2011-2013 (3 years)	2670	0.044	0.987	0.924	0.012	0.884
2011	991	0.045	0.986	0.917	0.012	0.883
2012	978	0.046	0.986	0.915	0.012	0.883
2013	715	0.039	0.990	0.938	0.011	0.885

All chi-square tests have 1 degree of freedom (df). RMSEA, Root mean squared error of approximation; CFI, Comparative fit index; TLI, Tucker-Lewis index; SRMR, Standardized root mean squared residual; CD, Coefficient of determination

**Table 5:** Multi-group confirmatory factor analysis for measurement invariance across years, sex, and age subgroups

Models	χ^2^ (df)	RMSEA	CFI	TLI	SRMR	CD	ΔCFI
BRFSS 2001-2010 (year subgroups)							
Full configural invariance	5430 (10)	0.041	0.987	0.925	0.011	0.88	−
Full metric invariance	5648 (37)	0.022	0.987	0.979	0.012	0.88	0
Full scalar invariance	10107 (64)	0.022	0.977	0.978	0.014	0.879	0.01
BRFSS 2011-2013 (year subgroups)							
Full configural invariance	2685 (3)	0.044	0.987	0.923	0.012	0.884	−
Full metric invariance	2696 (9)	0.025	0.987	0.974	0.012	0.884	0
Full scalar invariance	2794 (15)	0.02	0.987	0.984	0.012	0.884	0
BRFSS 2001-2010 (male vs. female)							
Full configural invariance	10443 (2)	0.057	0.997	0.981	0.01	0.879	−
Full metric invariance	12243 (5)	0.039	0.996	0.991	0.012	0.879	0.001
Full scalar invariance	23654 (8)	0.043	0.993	0.989	0.019	0.881	0.003
BRFSS 2011-2013 (male vs. female)							
Full configural invariance	5159 (2)	0.06	0.997	0.98	0.011	0.884	−
Full metric invariance	5788 (5)	0.041	0.996	0.991	0.012	0.884	0.001
Full scalar invariance	9425 (8)	0.041	0.994	0.991	0.017	0.885	0.002
BRFSS 2001-2010 (young vs. old ages)							
Full configural invariance	3587 (2)	0.034	0.999	0.994	0.007	0.877	−
Partial metric invariance	13086 (3)	0.053	0.996	0.984	0.014	0.87	0.003
Full metric invariance	62559 (5)	0.089	0.981	0.955	0.038	0.866	0.018
BRFSS 2011-2013 (young vs. old ages)							
Full configural invariance	2256 (2)	0.04	0.999	0.991	0.008	0.883	−
Partial metric invariance	7096 (3)	0.058	0.996	0.982	0.013	0.879	0.003
Full metric invariance	29614 (5)	0.092	0.981	0.955	0.035	0.874	0.018

Full configural invariance – same factor structure across groups; full metric invariance-equivalent factor loadings across groups; full scalar invariance – equivalent intercepts across groups; partial metric invariance – equivalent factor loadings across groups except factor loadings for mentally unhealthy day item. Age subgroups: young (18-64) and old (65+)

We also further assessed model stabilities across sex and age subgroups (Table 5). Results suggest that the one-factor model has strong measurement invariance across sex, holding full equivalent factor patterns, full equivalent factor loadings, and full equivalence intercepts between male and female adults. When applied to young (18-64) and old (65+) age subgroups, the one-factor model has full configural invariance but the full equivalent factor loadings is not supported as the CFI decrease is larger than 0.01. However, after releasing the equivalent factor loading constraints for the mentally unhealthy day item, partial metric invariance is tenable.

### Model application: trend monitoring

**Fig. 3:**
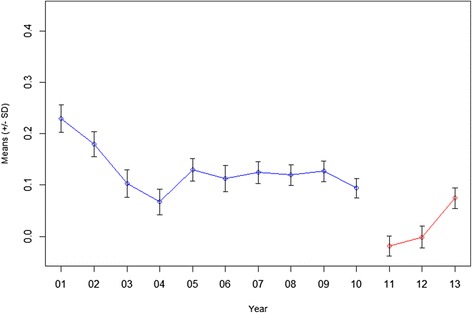
Trend analysis of overall HRQOL, BRFSS 2001–2010 and 2011–2013. The weighted and age-adjusted HRQOL summary scores were predicated by the model for the 2001–2010 and 2011–2013 periods, respectively. The mean HRQOL summary score for each year is shown from 2001 to 2013. The 2000 US Census population was used for age standardization.

The one-factor HRQOL model exhibits strong measurement invariance across year subgroups, which allows us to analyze how the mean of HRQOL factor scores changes over years. Figure 3 shows the age-standardized weighted means of HRQOL factor scores predicted for the 2001–2010 and 2011–2013 periods, respectively. The overall HRQOL scores gradually declined from 2001 to 2004 and, in general, remained stable thereafter through 2010 (*p* < 0.001 for 2001 vs. 2004, adjusted Wald test). Compared with 2011 and 2012, the overall HRQOL scores increased in 2013 (*p* < 0.001 for 2011 vs. 2013, adjusted Wald test). These findings were also confirmed with the changes from the original CDC HRQOL-4 questions (Additional file 1 shows results of CDC HRQOL-4 changes for 2001 vs. 2004, and 2011 vs. 2013).

## Discussion

In this study, we developed and tested a one-factor HRQOL model using a series of BRFSS data sets. To our knowledge, this is the first report of an HRQOL factor analysis based solely on CDC HRQOL-4. Two previous studies, which used data obtained from the optional BRFSS module, proposed a two-factor model [7, 17]. One report used summed z-scores from all items to represent physical and mental health, respectively. However, it did not consider item factor loadings and removed one item due to the cross loading issues [17]. As the CDC HRQOL-4 questions are more commonly used in BRFSS and other surveys, we performed HRQOL factor analysis using only these four items. EFA revealed that the four items could be explained by one underlying factor—a general health factor that encompasses both physical and mental health. As a result, this model could be used to generate a one-factor score that represents the underlying construct of HRQOL.

In addition to EFA, we performed CFA to evaluate our one-factor model with more statistical options such as goodness of fit, modification indices, and measurement invariance tests. Our post-hoc analysis found a negative error correlation path between the physically unhealthy day item and the mentally unhealthy day item. This result may have not only statistical support but also theoretical meaning. First, research has found that using similar question formats can affect survey responses [29]. The format of the two questions is very similar, which may contribute to the covariance between the two items. Second, our preliminary analysis (not shown) found that some individuals report no physically unhealthy days, but 30 mentally unhealthy days. Our one-factor model may account for this distinction by indicating a negative relationship between the error terms in the measures of physically unhealthy days and mentally unhealthy days.

Our one-factor model showed strong measurement invariance across year and sex subgroups. However, for young and old age subgroups, only partial metric invariance was observed due to different factor loadings on the mentally unhealthy day item. This may suggest that young and old people have different dimensions on mental health aspect, which is in accordance with previous reports [30, 31]. Further studies are needed to show how stable the factor structure is with other demographics, socioeconomic characteristics, and chronic conditions.

Using BRFSS 2001–2010 and 2011–2013 data sets, we demonstrated that our one-factor HRQOL model is stable over time, and could be used to monitor trends in HRQOL with a single summary score. This approach would be simpler, more comprehensive, and more representative than using the four individual CDC HRQOL-4 items. Using this new measure, we found that overall HRQOL decreased in the US from 2001 to 2014. This trend may have started even earlier: an analysis of data from BRFSS and NHANES from 1993 to 2001 also found gradual decreases in health-related quality of life among adults, as indicated by several measures [11].

This study has several limitations. First, our measures of HRQOL were based solely on CDC’s four core questions, which provide limited details about mental health symptoms. Second, the CDC HRQOL-4 questions are ordinal variables, which may have resulted in lower variance than would have existed had the variable been continuous. Thirdly, due to the large sample size, the chi-square test was not appropriate for our goodness of fit and model stability analyses. (The use of large samples can lead to significant p-values even if differences are small and meaningless. [32].) Instead, we used a list of other suitable statistics, such as RSMEA, CFI, and SMRM, to support our conclusions. Lastly, the study used self-reported data from BRFSS, which is subject to recall and social desirability biases, as well as non-response bias due to the exclusion of persons not living in a private residence.

Our model has several advantages: (1) it can be broadly used by public health professionals, as the CDC HRQOL-4 questions are included in several national survey systems including BRFSS and NHANES; (2) it provides one-factor score values that could represent HRQOL at both the individual and population levels; and (3) it exhibits strong measurement invariance or stability over time, which makes it suitable for trend monitoring. Public health professionals may also apply similar factor analyses to other state- or community-level data sets for local health research, assessment, and evaluation. Finally, though our analysis indicates the value of a summary factor score for overall HRQOL, the collection and application of the CDC-HRQOL-4 items still remain to be important for studying HRQOL, especially when focusing on more specific aspects of HRQOL (e.g., physical health or mental health).

## Conclusion

This study developed and tested a one-factor HRQOL model based on the CDC HRQOL-4 core questions. Using BRFSS data sets from 2001 to 2013, we evaluated the new model’s goodness of fit, validity, stability, and measurement invariance over time. We also demonstrated the application of the predicated HRQOL factor score in trend analysis. These results suggest that it is feasible to apply the CDC HRQOL-4 core questions to study HRQOL through factor analysis with one underlying construct. The resulting summary score of HRQOL may be applied to health evaluation, subgroup targeting, trend monitoring, and risk factor identification.

## Additional file

Additional file 1: Weighted Prevalences of Health-related Quality of Life Measures by Year, BRFSS. (DOCX 20 kb)
