# Supplementary material for: Summarizing health-related quality of life (HRQOL): development and testing of a one-factor model
Source: Popul Health Metr. 2016 Jul 11;14:22. doi: 10.1186/s12963-016-0091-3 (PMC4940947; doi:10.1186/s12963-016-0091-3)
Supplement: Additional file 1: — Weighted Prevalences of Health-related Quality of Life Measures by Year, BRFSS. (DOCX 20 kb) [file 12963_2016_91_MOESM1_ESM.docx]

| **Additional file 1 Weighted Prevalences of Health-related Quality of Life Measures by Year, BRFSS** | | | | | | | | | |
| --- | --- | --- | --- | --- | --- | --- | --- | --- | --- |
| **Year** | **2001** | | | | **2004** | | | |  |
| **HRQOL Measures** | **n** | **%** | **(95% CI)** | **n** | | **%** | **(95% CI)** | **P-value ^e^** | |
| **Fair/Poor Health ^a^** | 34,522 | 15.7 | (15.5-16.0) | 54,320 | | 16.4 | (16.2-16.7) | <0.001 | |
| **Frequent Physical Distress ^b^** | 27,049 | 11.8 | (11.6-12.0) | 43,211 | | 12.4 | (12.2-12.6) | <0.001 | |
| **Frequent Mental Distress ^c^** | 25,463 | 11.3 | (11.1-11.6) | 36,878 | | 11.8 | (11.6-12.0) | 0.008 | |
| **Frequent Activity Limitations ^d^** | 15,792 | 6.9 | ( 6.7- 7.1) | 25,816 | | 7.5 | ( 7.3- 7.7) | <0.001 | |
| **Year** | **2011** | | | **2013** | | | |  | |
| **HRQOL Measures** | **n** | **%** | **(95% CI)** | **n** | | **%** | **(95% CI)** | **P-value ^e^** | |
| **Fair/Poor Health ^a^** | 98,970 | 18.1 | (17.9-18.4) | 94,609 | | 18.2 | (17.9-18.4) | 0.938 | |
| **Frequent Physical Distress ^b^** | 80,307 | 14.1 | (14.0-14.3) | 76,961 | | 13.9 | (13.7-14.1) | 0.088 | |
| **Frequent Mental Distress ^c^** | 63,480 | 13.6 | (13.4-13.8) | 59,106 | | 13.0 | (12.8-13.2) | <0.001 | |
| **Frequent Activity Limitations ^d^** | 50,848 | 9.4 | ( 9.2- 9.6) | 48,744 | | 9.2 | ( 9.0- 9.3) | 0.037 | |
| ^a^ Self-reported health status. | | | | | |  |  |  | |
| ^b^ Physically unhealthy days ≥14 in the past 30 days. | | | | | |  |  |  | |
| ^c^ Mentally unhealthy days ≥14 in the past 30 days. | | | | | |  |  |  | |
| ^d^ Activity limitation days (due to poor physical or mental health) ≥14 in the past 30 days. Activity limitation days are zero when respondents have neither physically unhealthy days nor mentally unhealthy days. | | | | | | | | | |
| ^e^ Adjusted Wald test to compare prevalences between years. | | | | | |  |  |  | |
